# The efficacy of N-Acetylcysteine in severe COVID-19 patients: A structured summary of a study protocol for a randomised controlled trial

**DOI:** 10.1186/s13063-021-05242-4

**Published:** 2021-04-12

**Authors:** Arash Rahimi, Hamid Reza Samimagham, Mehdi Hassani Azad, Dariush Hooshyar, Mohsen Arabi, Mitra KazemiJahromi

**Affiliations:** 1 Clinical Research Development Center, Shahid Mohammadi Hospital, Hormozgan University of Medical Sciences, Bandar Abbas, Iran; 2 Infectious and Tropical Diseases Research Center, Hormozgan Health Institute, Hormozgan University of Medical Sciences, Bandar Abbas, Iran; 3 Student Research Committee, Faculty of Medicine, Hormozgan University of Medical Sciences, Bandar Abbas, Iran; 4 Department of Internal Medicine and Public Health Research Center, Family Medicine Department, Iran University of Medical Sciences, Tehran, Iran; 5 Endocrinology and Metabolism Research Center, Hormozgan University of Medical Sciences, Bandar Abbas, Iran

**Keywords:** COVID-19, Randomised controlled trial, Protocol, N-acetylcysteine

## Abstract

**Objectives:**

Severe acute respiratory infection (SARI) caused by the SARS-CoV-2 virus may cause lung failure and the need for mechanical ventilation. Infection with SARS-COV-2 can lead to activation of inflammatory factors, increased reactive oxygen species, and cell damage. In addition to mucolytic effects, N-Acetylcysteine has antioxidant effects that we believe can help patients recover. In this study, we evaluate the efficacy of N-Acetylcysteine in patients with severe COVID-19.

**Trial design:**

This is a prospective, randomized, single-blinded, phase 3 controlled clinical trial with two arms (ratio 1:1) parallel-group design of 40 patients, using the placebo in the control group.

**Participants:**

All severe COVID-19 patients with at least one of the following five conditions: (respiration rate > 30 per minute), hypoxemia (O2 ≤ saturation, arterial oxygen partial pressure ratio <300), pulmonary infiltration (> 50% of lung area during 24 48 h), Lactate dehydrogenase (LDH) > 245 U / l, Progressive lymphopenia, and admitted to the intensive care unit of Shahid Mohammadi Hospital in Bandar Abbas and have positive PCR test results for SARS-Cov-2 and sign the written consent of the study will be included. Patients will be excluded from the study if they have a history of hypersensitivity to N-Acetylcysteine, pregnancy, or refuse to participate in the study.

**Intervention and comparator:**

After randomization, participants in the intervention group receive standard of care (SOC) according to the National Committee of COVID-19 plus N-acetylcysteine (EXI-NACE 200mg/mL, in 10mL ampules of saline for parenteral injection (EXIR pharmaceutical company)) at a dose of 300 mg/kg equivalent to 20 gr as a slow single intravenous injection on the first day of hospitalization. In the control group patients receive SOC and placebo ( Sterile water for injection as the same dose). The placebo is identical in appearance to the N-acetylcysteine injection (EXIR pharmaceutical company as well).

**Main outcomes:**

The primary endpoint for this study is a composite endpoint for the length of hospitalization in the intensive care unit and the patient's clinical condition. These outcomes were measured at the baseline (before the intervention) and on the 14th day after the intervention or on the discharge day.

**Randomisation:**

Eligible participants (40) will be randomized in two arms in the ratio of 1: 1 (20 per arm) using online web-based tools and by permuted block randomization method. To ensure randomization concealment, random sequence codes are assigned to patients by the treatment team at the time of admission without knowing that each code is in the intervention or comparator group.

**Blinding (masking):**

All participants will be informed about participating in the study and the possible side effects of medication and placebo. Patients participating in the study will not be aware of the assignment to the intervention or control group. The principal investigator, health care personnel, data collectors, and those evaluating the outcome are aware of patient grouping.

**Numbers to be randomised (sample size):**

A total of 40 patients participate in this study, which are randomly divided; 20 patients in the intervention group will receive SOC and N-acetylcysteine, 20 patients in the control group will receive SOC and placebo.

**Trial status:**

First version of the protocol was approved by the Deputy of Research and Technology and the ethics committee of Hormozgan University of Medical Sciences on February 14, 2021, with the local code 990573, and the recruitment started on March 2, 2021 and the expected recruitment end date is April 1, 2021.

**Trial registration:**

The protocol was registered before starting participant recruitment entitled: Evaluation of the efficacy of N-Acetylcysteine in severe COVID-19 patients: a randomized controlled phase III clinical trial, IRCT20200509047364N3, at Iranian Registry of clinical trials on 20 February 2021.

**Full protocol:**

The full protocol is attached as an additional file, accessible from the Trials website (Additional file 1). In the interest in expediting dissemination of this material, the familiar formatting has been eliminated; this Letter serves as a summary of the key elements of the full protocol.

The study protocol has been reported in accordance with the Standard Protocol Items: Recommendations for Clinical Interventional Trials (SPIRIT) guidelines (Additional file 2).

**Supplementary Information:**

The online version contains supplementary material available at 10.1186/s13063-021-05242-4.

## Supplementary Information

**Additional file 1.** Full Study Protocol.

**Additional file 2.** SPIRIT 2013 Checklist: Recommended items to address in a clinical trial protocol and related documents.

## Data Availability

The authors have not still decided on the sharing of data.

