# Supplementary material for: The efficacy of N-Acetylcysteine in severe COVID-19 patients: A structured summary of a study protocol for a randomised controlled trial
Source: Trials. 2021 Apr 12;22:271. doi: 10.1186/s13063-021-05242-4 (PMC8040363; doi:10.1186/s13063-021-05242-4)
Supplement: Supplementary file 1 — Additional file 1. Full Study Protocol. [file 13063_2021_5242_MOESM1_ESM.pdf]

Title:

The efficacy of N-Acetylcysteine in severe COVID-19 patients: A structured summary of a study protocol for a randomised controlled trial

Authors

Arash Rahimi[1] , Hamid Reza Samimagham[2], Mehdi Hassani Azad [3], Dariush Hooshyar[4] , Mohsen Arabi[5], Mitra KazemiJahromi [6, *]

[1,2] Clinical Research Development Center, Shahid Mohammadi Hospital, Hormozgan University of Medical Sciences, Bandar Abbas, Iran

[3] Infectious and Tropical Diseases Research Center,Hormozgan Health Institute, Hormozgan University of Medical Sciences, Bandar Abbas, Iran

[4] Student Research Comitte,Faculty of Medicie, , Hormozgan University of Medical Sciences, Bandar Abbas, Iran

[5] Department of Internal Medicine and Public Health Research Center,Family Medicine Department,Iran University of Medical Sciences,Tehran, Iran

[6] Endocrinology and Metablism Research Center, Hormozgan University of Medical Sciences, Bandar Abbas, Iran

*** Correspondance:**

Mitra KazemiJahromi

Endocrinology and Metablism Research Center, Hormozgan University of Medical Sciences, Bandar Abbas, Iran

Tel: +989177912820*

Email: arashrahimi.article@gmail.com[1] , samimagham@yahoo.com[2] , mehdihassaniazad@gmail.com[3] ، dariush.hooshyar@gmail.com[4] , Arabi.m@iums.ac.ir[5] , , mitra.kazemijahromi@gmail.com[6] *

Abstract

Objectives

Severe acute respiratory infection (SARI) caused by the SARS-CoV-2 virus may cause lung failure and the need for mechanical ventilation. Infection with SARS-COV-2 can lead to activation of inflammatory factors, increased reactive oxygen species, and cell damage. In addition to mucolytic effects, N-Acetylcysteine has antioxidant effects that we believe can help patients recover. In this study, we evaluate the efficacy of N-Acetylcysteine in patients with severe COVID-19.

Trial design

This is a prospective, Concealed, randomized, single-blinded, phase 3 controlled clinical trial with two arms (ratio 1:1) parallel-group design of 40 patients, using the placebo in the control group.

Participants

All severe COVID-19 patients with at least one of the following five conditions:(respiration rate> 30 per minute), hypoxemia (O2 ≤ saturation, arterial oxygen partial pressure ratio <300), pulmonary infiltration (> 50% of lung area during 24 48 h), LDH> 245 U / l, Progressive lymphopenia, and admitted to the intensive care unit of Shahid Mohammadi Hospital in Bandar Abbas and have positive PCR test results for SARS-Cov-2 and sign the written consent of the study will be included. Patients will be excluded from the study if they have a history of hypersensitivity to N-Acetylcysteine, pregnancy, or refuse to participate in the study.

Intervention and comparator

After randomization, participants in the intervention group receive standard of care (SOC) according to the National Committee of COVID-19 plus N-acetylcysteine (Exi-Nac 2g / 10ml AMP (EXIR pharmaceutical company)) at a dose of 300 mg/kg equivalent to 20 gr as a slow single intravenous injection on the first day of hospitalization. in the control group patients receive SOC and placebo as the same dose.

Main outcomes

The primary endpoint for this study is a composite endpoint for the length of hospitalization in the intensive care unit and the patient's clinical condition. These outcomes were measured at the baseline (before the intervention) and on the 14th day after the intervention or on the discharge day.

Randomisation

Eligible participants (40) will be randomized in two arms in the ratio of 1: 1 (20 per arm) using online web-based tools and by permuted block randomization method. To ensure randomization concealment, random sequence codes are assigned to patients by the treatment team at the time of admission without knowing that each code is in the intervention or comparator group.

Blinding (masking)

All participants will be informed about participating in the study and the possible side effects of medication and placebo. Patients participating in the study will not be aware of the assignment to

the intervention or control group. The principal investigator, health care personnel, data collectors, and those evaluating the outcome are aware of patient grouping.

Numbers to be randomised (sample size)

A total of 40 patients participate in this study, which are randomly divided; 20 patients in the intervention group will receive SOC and N-acetylcysteine, 20 patients in the control group will receive SOC and placebo.

Keywords

COVID-19, Randomised controlled trial, protocol, N-acetylcysteine

Introduction:

Severe acute respiratory infection (SARI) caused by the SARS-CoV-2 virus may lead to lung failure and the need for mechanical ventilation. (1–3)

The mechanisms by which the virus affects the alveolar epithelium are not fully understood. (4) However, upon reaching the lower airways, the virus Spike protein appears to bind to the angiotensin-converting enzyme 2 (ACE2) and use it as a vehicle to enter alveolar cells. (5)

ACE2 is an enzyme that catalyzes the conversion of angiotensin II (AngII) to angiotensin 1-7 and appears to be inactivated by viral function. (6)

Physiological intracellular signaling of AngII involves increased production of reactive oxygen species (ROS), either through the activity of the Nox enzyme (7) or mitochondria. (8) Initially, these ROSs are used in signaling mechanisms; however, their excessive levels can lead to apoptosis or cell necrosis. (9)

Also, vascular smooth muscle cells in tissue culture exposed to AngII increase CD40 expression, which is an important mediator in the acquired immune response. (10) This phenomenon is reduced through an oxidation signaling pathway, which involves increasing Nox expression and hydrogen peroxide production. In some experiments, AngII-induced CD40 expression was blocked by N-acetylcysteine (NAC) treatment. (10)

NAC has been used clinically as a mucolytic since the 1960s. (11) Also currently used in acute liver failure or acetaminophen poisoning. (12,13) Its safety is well documented and its effectiveness in lung diseases may go beyond its mucolytic function, as it may also interfere with the inflammatory response and bronchial tone. (14)

NAC may also replenish intracellular glutathione (GSH) reservoirs by preparing cysteine, a precursor essential for GSH synthesis. (15) Thus, NAC administration can restore the primary intracellular antioxidant system and intracellular oxidation signaling by increasing decreased activity (GSH-GSSG). (16)

Therefore, we designed a single-blind, controlled randomized clinical trial to answer the question: (Can NAC be effective in severe COVID-19 patients?).

Methode:

This study is a phase 3 randomized clinical trial, with the grouping of two parallel arms on 40 patients and the use of placebo in the control group, single-blinded, and randomization concealment that has been registered with the code IRCT20200509047364N3 to the Iranian Clinical Trial Registration Center (IRCT). This study has also been approved by the Research Ethics Committee of Hormozgan University of Medical Sciences with the code IR.HUMS.REC.1399.539.

Data collection and recruitment is done in Shahid Mohammadi Hospital in Bandar Abbas. (Email: Shmh@hums.ac.ir, Web page: https://shmh.hums.ac.ir/)

Eligibility criteria:

Inclusion criteria: All COVID-19 patients whose disease has been confirmed by the PCR test for SARS-Cov-2. Having one of the criteria for severe COVID-19 disease includes tachypnea (respiration rate> 30 per minute), hypoxemia (O2 ≤ saturation, arterial oxygen partial pressure ratio <300), pulmonary infiltration (> 50% of lung area during 24 48 h), LDH> 245 U / l, Progressive lymphopenia. Hospitalized in the intensive care unit. Signing the written consent of the study participant.

Exclusion criteria: Known allergy or hypersensitivity to N-Acetylcysteine. Pregnancy The participant refused to participate in the continuation of the study.

Randomization:

Before assigning groups to individuals eligible to participate in the study, informed consent is completed for grouping individuals. the person who has no role in admitting patients and assigning patients to random codes preparing random sequences using online tools (https://www.sealedenvelope.com/) and by permuted block randomization method. Individualized random allocation is done in blocks with sizes 2 and 4, and without stratification. eligibility criteria are monitored by the person responsible for admitting patients. Codes in a random sequence are assigned to patients by the treatment team without knowing that each code is in the intervention or placebo group. Patient codes are then matched to randomly generated sequence information for interventions. (randomization concealment is done by the treatment team without informing the person responsible for admitting patients and the person who prepared the random sequence.)

Blinding description:

In this study, all participants are aware of participating in this study and enter the study with their consent. All participants are unaware of which group of this study they are in and after grouping

patients in the groups, Patients receive N-Acetylcysteine in the treatment group and receive a placebo in the control group. The lead researcher, health care personnel, data collection officials, and those who evaluate the outcome are aware of the grouping of patients. Those who prepare the draft of the article are unaware of the groupings if they do not cooperate in the above cases.

Sample size:

Due to the lack of previous studies, the sample size of 40 patients who were divided into two groups of ten for intervention and control was used.

Outcomes and measurement:

Data collection is done by the medical team of Shahid Mohammadi Hospital and patients' records.

Primary outcomes:

The length of hospitalization of patients in the intensive care unit and the clinical condition of patients are considered as the primary outcomes of the study. The measurement of these outcomes is by using the information of patients during the hospitalization and the opinion of the treating physician. All primary outcomes were assessed At the beginning of the study (before the intervention) and day 14 after the intervention or the day of the patient's discharge.

Secondary outcomes:

Respiratory rate and Oxygen saturation state measured by Pulse oximeter, Lung infiltration status measured by Chest X-ray, Lactate Dehydrogenase (LDH) levels, C-reactive protein (CRP) level's, Lymphocyte count, and Platelet count measured by Pathobiology laboratory. All secondary outcomes were assessed At the beginning of the study (before the intervention) and day 14 after the intervention or the day of the patient's discharge.

Intervention groups:

Intervention group:

The treatment group receives standard drug therapy based on the treatment protocols of the National Committee COVID-19 and N-Acetylcysteine (Exi-Nac 2g/10ml AMP (EXIR pharmaceutical company)) at a dose of 300 mg/kg equivalent to 20 g as a slow single intravenous injection on the first day of hospitalization. Vital signs of patients are also checked at regular intervals and frequently. Standard pharmacotherapy according to the treatment protocols of the National Committee of COVID-19 includes Hydroxychloroquine / Chloroquine Phosphate: Hydroxychloroquine sulfate tablets 200 mg or chloroquine phosphate tablets 250 mg (equivalent

to 150 mg base dose) 2 tablets every 12 hours on the first day and then one tablet every 12 hours for at least 7 days and up to 14 days. One of the following medications at the discretion and diagnosis of the treating physician: kaletra tablets (Lopinavir / Ritonavir) 50/200 mg every 12 hours 2 pieces after meals for at least 7 days and a maximum of 14 days. Tablets (Atazanavir / Ritonavir) 300/100 One tablet daily with food or Atazanavir 400 mg daily for at least 7 days and up to 14 days.

Control group:

The placebo group receives standard drug therapy based on the treatment protocols of the National COVID-19 Committee and placebo as a slow single intravenous injection on the first day of hospitalization. Standard pharmacotherapy according to the treatment protocols of the National Committee of COVID-19 includes Hydroxychloroquine / Chloroquine Phosphate: Hydroxychloroquine sulfate tablets 200 mg or chloroquine phosphate tablets 250 mg (equivalent to 150 mg base dose) 2 tablets every 12 hours on the first day and then one tablet every 12 hours for at least 7 days and up to 14 days. One of the following medications at the discretion and diagnosis of the treating physician: kaletra tablets (Lopinavir / Ritonavir) 50/200 mg every 12 hours 2 pieces after meals for at least 7 days and a maximum of 14 days. Tablets (Atazanavir / Ritonavir) 300/100 One tablet daily with food or Atazanavir 400 mg daily for at least 7 days and up to 14 days.

Statistical analysis:

IBM-SPSS version 22 software will be used for data analysis, independent t-test and Mann-Whitney will be used to compare the means of quantitative data.

Chi-square and Fisher's test were used to compare qualitative variables.

Trial Status:

First version of the protocol was approved by the Deputy of Research and Technology and the ethics committee of Hormozgan University of Medical Sciences on February 14, 2021, with the local code 990573, and the recruitment started on March 2, 2021 and its continues. Expected recruitment end date is April 1, 2021.

Trial registration:

The protocol was registered before starting subject recruitment under the title: Evaluation of the efficacy of N-Acetylcysteine in severe COVID-19 patients: a randomized controlled phase III clinical trial, at Iranian Registry of clinical trials (https://www.irct.ir) on 20 February 2021.

Declarations

Ethics approval and consent to participate

The protocol was approved by the ethics committee of Hormozgan University of Medical Sciences on February 14, 2021, with the code IR.HUMS.REC.1399.539. (https://ethics.research.ac.ir/EthicsProposalView.php?id=180568)

The authors confirm that this trial has received ethical approval from the appropriate ethical committee as described above. Written prospective informed consent will be obtained from participants before involvement in the trial in the Persian language.

Consent for publication

Written informed consent will be obtained from all participants/subject's legally acceptable representatives before inclusion in the trial for collecting data, analysis, storage, and publishing it.

Availability of data and materials

The authors have not still decided on the sharing of data.

Competing interests

The authors declare that they have no competing interests.

Funding

This study is supported by the Deputy of Research and Technology of Hormozgan University of Medical Sciences; Also, this sponsor has no role in designing the study, collection, storage, and analysis of information and in preparing the manuscript.

Authors' contributions

M KJ. and A R. designed the study. All the authors contributed in data collection and manuscript writing. M KJ supervised the study. The author(s) read and approved the final manuscript.

Acknowledgements

This work was a dissertation supported by the Clinical Research Development Center of Shahid Mohammadi Hospital. Bandar Abbas. Iran. We are sincerely thankful to Miss Tayyebeh Zaree in the Clinical Research Development Center of Shahid Mohammadi Hospital.

Authors' information (optional)

Affiliations:

Clinical Research Development Center, Shahid Mohammadi Hospital, Hormozgan University of Medical Sciences, Bandar Abbas, Iran

Arash Rahimi & Hamid Reza Samimagham

Infectious and Tropical Diseases Research Center, Hormozgan Health Institute, Hormozgan University of Medical Sciences, Bandar Abbas, Iran

Mehdi Hassani Azad

Student Research Committee, Faculty of Medicine, Hormozgan University of Medical Sciences, Bandar Abbas, Iran

Dariush Hooshyar

Department of Internal Medicine and Public Health Research Center, Family Medicine Department, Iran University of Medical Sciences, Tehran, Iran

Mohsen Arabi

Endocrinology and Metabolism Research Center, Hormozgan University of Medical Sciences, Bandar Abbas, Iran

Mitra KazemiJahromi

References:

1. Richardson S, Hirsch JS, Narasimhan M, Crawford JM, McGinn T, Davidson KW, et al. Presenting Characteristics, Comorbidities, and Outcomes Among 5700 Patients Hospitalized With COVID-19 in the New York City Area. JAMA [Internet]. 2020 May 26 [cited 2020 Nov 27];323(20):2052. Available from: https://jamanetwork.com/journals/jama/fullarticle/2765184
2. Docherty AB, Harrison EM, Green CA, Hardwick HE, Pius R, Norman L, et al. Features of 20 133 UK patients in hospital with covid-19 using the ISARIC WHO Clinical Characterisation Protocol: Prospective observational cohort study. The BMJ [Internet]. 2020 May 22 [cited 2020 Nov 27];369. Available from: https://isaric4c.net
3. Grasselli G, Zangrillo A, Zanella A, Antonelli M, Cabrini L, Castelli A, et al. Baseline Characteristics and Outcomes of 1591 Patients Infected with SARS-CoV-2 Admitted to ICUs of the Lombardy Region, Italy. JAMA - Journal of the American Medical Association [Internet]. 2020 Apr 28 [cited 2020 Nov 27];323(16):1574–81. Available from: https://jamanetwork.com/
4. Tay M, Poh C, Rénia L, … PM-NR, 2020 undefined. The trinity of COVID-19: immunity, inflammation and intervention. nature.com [Internet]. [cited 2020 Nov 27]; Available from: https://www.nature.com/articles/s41577-020-0311-8?fbclid=IwAR006BPp8mRx8nAxiOljiN8dlYJjNncQ4zj4N8mvi1VN9gMFrcSRuIpnTWk
5. Hoffmann M, Kleine-Weber H, Schroeder S, Krüger N, Herrler T, Erichsen S, et al. SARS-CoV-2 Cell Entry Depends on ACE2 and TMPRSS2 and Is Blocked by a Clinically Proven Protease Inhibitor. Cell. 2020 Apr 16;181(2):271-280.e8.
6. Touyz RM, Li H, Delles C. ACE2 the Janus-faced protein-from cardiovascular protection to severe acute respiratory syndrome-coronavirus and COVID-19 [Internet]. Vol. 134, Clinical Science. Portland Press Ltd; 2020 [cited 2020 Nov 27]. p. 747–50. Available from: /clinsci/article/134/7/747/222585/ACE2-the-Janus-faced-protein-from-cardiovascular
7. Laurindo FRM, de Souza HP, Pedro MDA, Janiszewski M. Redox aspects of vascular response to injury. Methods in Enzymology. 2002 Jan 1;352:432–54.
8. Dikalov SI, Nazarewicz RR. Angiotensin II-induced production of mitochondrial reactive oxygen species: Potential mechanisms and relevance for cardiovascular disease [Internet]. Vol. 19, Antioxidants and Redox Signaling. Mary Ann Liebert, Inc. 140 Huguenot Street, 3rd Floor New Rochelle, NY 10801 USA ; 2013 [cited 2020 Nov 27]. p. 1085–94. Available from: https://www.liebertpub.com/doi/abs/10.1089/ars.2012.4604
9. Redza-Dutordoir M, Averill-Bates DA. Activation of apoptosis signalling pathways by reactive oxygen species. Vol. 1863, Biochimica et Biophysica Acta - Molecular Cell Research. Elsevier B.V.; 2016. p. 2977–92.
10. Souza HP, Frediani D, Cobra AL, Moretti AI, Jurado MC, Fernandes TR, et al. Angiotensin II modulates CD40 expression in vascular smooth muscle cells. Clinical Science [Internet]. 2009 Mar 1 [cited 2020 Nov 27];116(5):423–31. Available from: /clinsci/article/116/5/423/68642/Angiotensin-II-modulates-CD40-expression-in

11. Ehre C, Rushton ZL, Wang B, Hothem LN, Morrison CB, Fontana NC, et al. An improved inhaled mucolytic to treat airway muco-obstructive diseases. American Journal of Respiratory and Critical Care Medicine [Internet]. 2019 Jan 15 [cited 2020 Nov 27];199(2):171–80. Available from: https://www.atsjournals.org/doi/10.1164/rccm.201802-0245OC

12. Lee WM, Hynan LS, Rossaro L, Fontana RJ, Stravitz RT, Larson AM, et al. Intravenous N-Acetylcysteine Improves Transplant-Free Survival in Early Stage Non-Acetaminophen Acute Liver Failure. Gastroenterology. 2009 Sep 1;137(3):856-864.e1.

13. Fisher ES, Curry SC. Evaluation and treatment of acetaminophen toxicity. In: Advances in Pharmacology. Academic Press Inc.; 2019. p. 263–72.

14. Cazzola M, Calzetta L, Page C, Rogliani P, Matera MG. Thiol-Based Drugs in Pulmonary Medicine: Much More than Mucolytics. Vol. 40, Trends in Pharmacological Sciences. Elsevier Ltd; 2019. p. 452–63.

15. Ershad M, Naji A, Vearrier D. N Acetylcysteine [Internet]. StatPearls. StatPearls Publishing; 2020 [cited 2020 Nov 27]. Available from: http://www.ncbi.nlm.nih.gov/pubmed/30725868

16. Oliveira PVS, Laurindo FRM. Implications of plasma thiol redox in disease [Internet]. Vol. 132, Clinical Science. Portland Press Ltd; 2018 [cited 2020 Nov 27]. p. 1257–80. Available from: /clinsci/article/132/12/1257/72113/Implications-of-plasma-thiol-redox-in-disease
